# PUTransGCN: identification of piRNA–disease associations based on attention encoding graph convolutional network and positive unlabelled learning

**DOI:** 10.1093/bib/bbae144

**Published:** 2024-04-05

**Authors:** Qiuhao Chen, Liyuan Zhang, Yaojia Liu, Zhonghao Qin, Tianyi Zhao

**Affiliations:** Institute of Bioinformatics, Harbin Institute of Technology, 150000, Harbin, Heilongjiang, China; School of Computer Science and Technology, Harbin Institute of Technology, 150000, Harbin, Heilongjiang, China; School of Computer Science and Technology, Harbin Institute of Technology, 150000, Harbin, Heilongjiang, China; State Key Laboratory of Robotics and System, Harbin Institute of Technology, 150000, Harbin, Heilongjiang, China; School of Computer Science and Technology, Harbin Institute of Technology, 150000, Harbin, Heilongjiang, China

**Keywords:** piRNA–disease associations, positive unlabelled learning, heterogeneous network, graph convolutional network, attention mechanism

## Abstract

Piwi-interacting RNAs (piRNAs) play a crucial role in various biological processes and are implicated in disease. Consequently, there is an escalating demand for computational tools to predict piRNA–disease interactions. Although there have been computational methods proposed for the detection of piRNA–disease associations, the problem of imbalanced and sparse dataset has brought great challenges to capture the complex relationships between piRNAs and diseases. In response to this necessity, we have developed a novel computational architecture, denoted as PUTransGCN, which uses heterogeneous graph convolutional networks to uncover potential piRNA–disease associations. Additionally, the attention mechanism was used to adjust the weight parameters of aggregation heterogeneous node features automatically. For tackling the imbalanced dataset problem, the combined positive unlabelled learning (PUL) method comprising PU bagging, two-step and spy technique was applied to select reliable negative associations. The features of piRNAs and diseases were derived from three distinct biological sources by PUTransGCN, including information on piRNA sequences, semantic terms related to diseases and the existing network of piRNA–disease associations. In the experiment, PUTransGCN performs in 5-fold cross-validation with an AUC of 0.93 and 0.95 on two datasets, respectively, which outperforms the other six state-of-the-art models. We compared three different PUL methods, and the results of the ablation experiment indicate that the combined PUL method yields the best results. The PUTransGCN could serve as a valuable piRNA–disease prediction tool for upcoming studies in the biomedical field. The code for PUTransGCN is available at https://github.com/chenqiuhao/PUTransGCN

## INTRODUCTION

Piwi-interacting RNAs (piRNAs) have gained substantial attention in the world of bioinformatics and health sciences due to their significant role in various biological processes and their associations with a myriad of diseases, including cancer, neurodegenerative disorders and other geriatric conditions [1–4]. Spanning about 24–35 nucleotides in length, piRNAs bind with Piwi-subfamily Argonaute proteins and are critical in the silencing of transposable elements, genome defence and histone modification, among other functions [5, 6].

The growing body of research linking the aberrant expression of piRNAs with diverse diseases underscores the potential of these small non-coding RNAs (ncRNAs) as diagnostic markers and therapeutic targets. For example, an increase was observed in 181 piRNAs and a decrease in 129 piRNAs within the cardiospheres when compared with cardiosphere-derived cells [7]. piR-39980, when overproduced in neuroblastoma cells, functions as an oncogenic piRNA, contributing to tumour development. Suppressing this piRNA led to a decrease in cell survival, invasive ability and the propensity for IMR-32 cells to spread [8]. One study shows a decrease in the expression of piRNAs (piR-1311, piR-16677, piR-20365, piR-4153) in leiomyomas when contrasted with normal myometrial tissue [9]. Analysis of the sequencing data revealed that a series of piRNAs, specifically piR-2660989, piR-10506469, piR-20548188, piR-10822895, piR-hsa-23209 and piR-18044111, demonstrated increased levels in the plasma samples from patients with cholangiocarcinoma and gallbladder carcinoma [10].

The advent of databases like piRDisease [11], NcRPheno [12] and MNDR [13] has provided a wealth of data for such associations [14]. piRDisease v1.0 has collected a database with 7939 associations that have been curated manually, linking 4796 piRNAs to 28 distinct diseases. It was used in many piRNA–disease association (PDA) prediction models including iPiDA-sHN [15], iPiDi-PUL [16], piRDA [17], SPRDA [18], ETGPDA [19], LGAT [20] and iPiDA-GBNN [21]. NcRPheno presents itself as an all-encompassing database dedicated to ncRNA–disease associations, confirming 1282 PDAs through experimental validation. Meanwhile, MNDR offers a platform to collate a variety of ncRNA–disease associations documented in biological research literature. iPiDA-GCN [22] and iPiDA-SWGCN [23] use MNDR v3.0 as training set.

Numerous PDAs have been confirmed through biological experiments to date. However, the progress in this area could be significantly hindered by the high investment of time and resources. In response to these challenges, researchers have developed a variety of computational algorithms to better navigate piRNA–disease networks. For the models using traditional machine learning method, iPiDi-PUL [16] and piRDA [17] encode each pair of associations by concatenating the features of each piRNA and disease, extract the main features through PCA and then classify unlabelled associations using machine learning methods such as random forest. They, respectively, use similarity matrix and one hot as the initial features of nodes, and both use positive unlabelled learning (PUL) method to select reliable negative associations.

Deep learning has commonly been applied in the field of bioinformatics. In contrast to conventional machine learning techniques, deep learning algorithms demonstrate enhanced non-linear fitting capabilities. For the nodes association prediction task, graph networks are effective in extracting potential topology features. iPiDA-GCN [22] and ETGPDA [19] use an integrated heterogeneous network of PDAs to aggregate the feature of different nodes. This method can better take into account the information between nodes compared with traditional machine learning methods. iPiDA-SWGCN [23] further addresses the sparsity issue of adjacency matrix through a weighted approach. GAPDA [20] allocates weights between nodes dynamically using line graph attention in heterogeneous network. With the help of gradient boosting techniques, iPiDA-GBNN [21] incrementally constructs complex GrowNet network using shallow networks to enhance the robustness of the model.

While the mentioned methods have demonstrated good performance, there is room for enhancement. On the one hand, the sequence information of piRNA has not been fully utilized, and a lot of information will be lost by only representing features through similarity matrix. On the other hand, the process of selecting reliable negative associations can be more refined.

To address these drawbacks, we proposed a model PUTransGCN based on attention encoding graph convolutional network and PUL. Our main contributions are summarized as follows:

- We use word2vec and TextCNN to embed the piRNA sequence, which not only includes information of the entire sequence but also information of subsequences.
- Three PUL methods including PU bagging, two-step and spy technique are applied to select reliable negative associations for tackling the problem of imbalanced dataset.
- Two attention modules are applied to integrate the feature of piRNAs and diseases. piRNAs and diseases act as query and key to calculate the attention score between them, which can capture more complex information between nodes comprehensively.
- Experimental results demonstrate that PUTransGCN outperforms other six state-of-the-art methods. Case studies show its predictive capability.

## MATERIALS

### Dataset

We evaluated our model in two datasets:

- MNDR 4.0 MNDR 4.0 [13] is a comprehensive ncRNA–disease database that integrates manual curation of over 40 000 published studies and 23 other experimentally validated databases. After removing duplicate and ambiguous associations, 9616 PDAs are collected, comprising 8205 piRNAs and 15 diseases. Known associations represent 7.81% of all associations.
- piRDisease v1.0 piRDisease v1.0 [11] is a piRNA–disease database that contains 7939 verified associations involving 4796 piRNAs that have been experimentally linked to 28 different diseases. 5002 PDAs are selected after removing duplicated data, comprising 4350 piRNAs and 21 diseases. Known associations account for 5.48% of the total associations.

## METHODOLOGY

**Figure 1:**
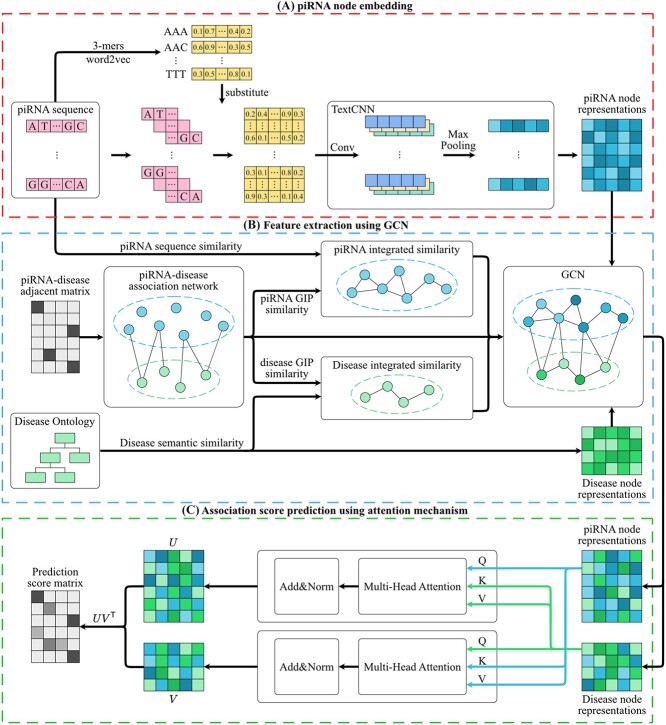
The flowchart of PUTransGCN. (**A**) piRNA node embedding. Split each piRNA sequence into several 3-mers, substitute each 3-mers by the feature vector obtained by word2vec, then embed piRNA node feature using TextCNN. (**B**) Feature extraction using GCN. Construct a heterogeneous network based on piRNA similarity matrix, disease similarity matrix and piRNA–disease adjacent matrix. Extract piRNA and disease features using GCN. (**C**) Association score prediction using the attention mechanism. For the encoder that extracts piRNA features, the $\boldsymbol{Q}$ matrix is the piRNA feature matrix and the $\boldsymbol{K}$, $\boldsymbol{V}$ matrices are the disease feature matrix. For the encoder that extracts disease features, it is opposite to this.

We proposed a predictor PUTransGCN based on graph attention network (GCN) and attention mechanism to predict PDAs; PUL was applied while training to avoid the imbalance dataset problem. The workflow of PUTransGCN is shown in Figure 1. There are mainly three steps: (a) Embedding piRNA sequence using the TextCNN technique. (b) Extracting the piRNA and disease feature using heterogeneous GCN. (c) Predicting association score using attention mechanism.

### piRNA sequence embedding

In most previous studies, the feature of a piRNA is commonly represented by the similarity with other piRNAs [15, 16, 18–20, 22, 23] or a one-hot vector [17]. However, the similarity obtained by the alignment algorithm can only measure the degree of matching for the entire sequence but loses the information implied in the local subsequence. For the one-hot embedding method, the disadvantage is that the one-hot vector is sparse, so it is difficult to describe the similarity between different piRNAs accurately.

To tackle these problems, we introduce an effective sequence embedding model DeepLncLoc proposed by Zeng *et al*. [24]. This method uses the 3-mer to encapsulate the compositional properties of sequences, which can reflect certain biological characteristics and patterns inherent to the molecule. Compared with the traditional k-mer features extraction method, which is only concerned with the occurrence of the k-mer and ignores the position of k-mer in the raw sequence, this method can keep the order information of the sequence and work well in the ncRNA subcellular localization prediction task. The main idea is to divide a piRNA sequence into non-overlapping subsequences and extract the patterns from each subsequence. The workflow of the piRNA embedding is shown in Figure 1(A). the steps of embedding a piRNA sequence are as follows:

1. Split all piRNA sequences into several consecutive 3-mer subsequences. The piRNA sequence is denoted as follows:

(1) \begin{align*}& \operatorname{piRNA}=S_{1}, ~S_{2}, \ldots, ~S_{m},\end{align*}

where $\mathrm{S}_{i}$ is the $i$th subsequence in the piRNA sequence. For example, a sequence ‘ATTGCAT’ can be divided as {ATT, TTG, TGC, GCA, CAT}.
2. Apply the word2vec technique to embed each 3-mer subsequence. Considering the whole sequence and 3-mer subsequence as sentence and word, respectively, the Word2vec technique was applied in the genism library (Rehurek, R. and Sojka) [25] to pre-train all piRNA sequences in the dataset. There are two kinds of popular word embedding models: skip-gram and continuous bag of words. In this study, we used the skip-gram model to encode each 3-mer feature into a vector because it improves the accuracy of the representations of less frequent words [26].
3. Encode each 3-mer feature by looking up the pre-trained vectors. Combine these vectors into a matrix representing the whole piRNA sequence.
4. Use TextCNN to extract high-level features of the sequence for integrating the information about different parts into a vector, which leverages multiple filters of varying sizes to extract salient features from the whole sequence [27], effectively capturing local correlations by applying three different convolutional kernels(sizes=1, 3, 5) over the sequence; a max pooling layer is then performed to get the most significant features and reduce the dimension. Figure shows the workflow of a piRNA sequence embedding.

### piRNA–disease heterogeneous network construction

piRNA–disease heterogeneous network is composed of three parts as follows: 


(2) \begin{align*}& \boldsymbol{A}_{h}=\begin{bmatrix} \boldsymbol{S}_{p} & \boldsymbol{A} \\ \boldsymbol{A}^{T} & \boldsymbol{S}_{d} \end{bmatrix},\end{align*}

where $\boldsymbol{S}_{p} \in \mathbb{R}^{m \times m}$ is the piRNA similarity matrix, $\boldsymbol{S}_{d} \in \mathbb{R}^{n \times n}$ is the disease similarity matrix, $\boldsymbol{A} \in \mathbb{R}^{m \times n}$ is the piRNA–disease adjacency matrix and $m$ and $n$ are the number of piRNAs and diseases, respectively.

#### PDA matrix

The adjacency matrix represents if there is an association between each piRNA and disease. It can be denoted as follows: 


(3) \begin{align*}& \boldsymbol{A} =\left[\begin{array}{@{}cccc@{}} a_{1,1} & a_{1,2} & \cdots & a_{1, n} \\ a_{2,1} & a_{2,2} & \cdots & a_{2, n} \\ \vdots & \vdots & \ddots & \vdots \\ a_{m, 1} & a_{m, 2} & \cdots & a_{m, n} \end{array}\right]\end{align*}

$a_{ij}=1$ if there is an association between $i$th piRNA and $j$th disease, and $a_{ij}=0$ otherwise.

#### piRNA similarity matrix

The similarity between piRNAs is obtained from the sequences’ information and the adjacency matrix, which is represented as $\boldsymbol{PS}_{\text{seq}}\in \mathbb{R}^{m\times m}$.

Based on the assumption that piRNAs with similar sequences are more likely to have similar functions, the Smith–Waterman alignment algorithm [28] is applied to evaluate the similarity between piRNAs. The sequences of piRNAs are obtained from piRBase v3.0 [http://bigdata.ibp.ac.cn/piRBase], which is a manually curated resource focused on assisting piRNA functional analysis [29]. After normalization, the formula for calculating sequence similarity is as follows: 


(4) \begin{align*}& S_{p}^{\text{seq}}(p_{i}, p_{j})=\frac{\operatorname{SW}(p_{i}, p_{j})}{\sqrt{\operatorname{SW}(p_{i}, p_{i}) \times \operatorname{SW}(p_{j}, p_{j})}},\end{align*}

where $\mathrm{SW}(p_{i}, p_{j})\in \mathbb{R}^{m\times m}$ represents the sequence alignment score between $i$th and $j$th piRNA based on the Smith–Waterman alignment algorithm.

Gaussian interaction profile (GIP) kernel similarity has been commonly used to evaluate the similarity between two nodes in the ncRNA–disease association prediction task [15, 16, 18–20], suggesting that similar piRNAs show similar interaction patterns in diseases and vice versa. The GIP kernel similarity between $i$th and $j$th piRNA is as follows [30]: 


(5) \begin{align*}& S_{p}^{\text{GIP}}(p_{i}, p_{j})=\exp \left(-\lambda_{p}\|\boldsymbol{A}(p_{i},)-\boldsymbol{A}(p_{j},)\|^{2}\right),\end{align*}

where $A(p_{i},)$ and $A(p_{j},)$ are the $i$th and $j$th row vector of the adjacency matrix $A$, and $\lambda _{p}$ is the kernel width coefficient, which is defined as 


(6) \begin{align*}& \lambda_{p}=\frac{1}{\frac{1}{N_{p}} \sum_{k=1}^{N_{p}}\left\|\boldsymbol{A}(p_{k}, )\right\|^{2}},\end{align*}

where $N_{p}$ is the number of total piRNA, $A(p_{k},)$ is the $k$-th row vector of the adjacency matrix $A$.

The integrated similarity between piRNAs is the mean of sequence similarity and GIP kernel similarity: 


(7) \begin{align*}& \boldsymbol{S}_{p}=\frac{\boldsymbol{S}_{p}^{\text{seq}}+\boldsymbol{S}_{p}^{\text{GIP}}}{2}\end{align*}

#### Disease similarity matrix

This study uses disease ontology (DO) [31] described in directed acyclic graph (DAG) [32] to compute the semantic similarity between diseases, which has been widely used in ncRNA–disease association identification [16, 19, 20, 22, 23]. DO is a standardized ontology for human disease, effectively representing the topological relationship between diseases. Based on the hierarchical relationship between diseases obtained from DO, the DAG-based algorithm calculates the semantic similarity between diseases, indicating that a more significant number of shared parent diseases implies higher similarity. The semantic similarity between the $i$th and $j$th disease are calculated as follows [33]: 


(8) \begin{align*}& S_{d}^{\text{sem}}\left(d_{i}, d_{j}\right)=\frac{\sum_{t \in T_{i} \cap T_{j}}\left(S_{d_{i}}(t)+S_{d_{j}}(t)\right)}{\sum_{t \in T_{i}} S_{d_{i}}(t)+\sum_{t \in T_{j}} S_{d_{j}}(t)},\end{align*}

where $T_{i}$ is the set including all diseases in the DAG of the $i$th disease, and $S_{d_{i}}$ represents the semantic impact that diseases $t \in T_{i}$ has on the $i$th disease; it is calculated as follows: 


(9) \begin{align*}& \left\{\begin{array}{@{}ll} S_{d_{k}}(t)=\max \left\{\theta \cdot S_{d_{k}}(t \prime) \mid t^{\prime} \in \text{children of}(t)\right\} & \text{if\ } d_{k} \neq d_{j} \\ S_{d_{k}}(t)=1 & \text{otherwise} \end{array}\right.,\end{align*}

where $\theta $ is the decaying parameter set as 0.5 [33]. The less the intersection between the parents of two diseases, the lower the semantic similarity.

Similar to piRNA, the GIP kernel similarity between diseases is as follows [30]: 


(10) \begin{align*}& S_{d}^{\text{GIP}}\left(d_{i}, d_{j}\right)=\exp \left(-\lambda_{d}\|\boldsymbol{A}(,d_{i})-\boldsymbol{A}(,d_{j})\|^{2}\right),\end{align*}

where $\boldsymbol{A}(,d_{i})$ and $\boldsymbol{A}(,d_{j})$ are the $i$th and $j$th column vector of the adjacency matrix $\boldsymbol{A}$, and $\lambda _{d}$ is the kernel width coefficient, which is defined as 


(11) \begin{align*}& \lambda_{d}=\frac{1}{\frac{1}{N_{d}} \sum_{k=1}^{N_{p}}\left\|\boldsymbol{A}(,d_{k})\right\|^{2}}\end{align*}

The disease similarity matrix is obtained by semantic similarity and GIP kernel similarity: 


(12) \begin{align*}& \boldsymbol{S}_{d}=\frac{\boldsymbol{S}_{d}^{\text{sem}}+\boldsymbol{S}_{d}^{\text{GIP}}}{2}\end{align*}

### Feature extraction using GCN

GCN has been widely adopted in network analysis tasks due to its ability to capture high-order graph relations and propagate information through the network [19, 22, 23], which is a type of neural network that can effectively extract features from heterogeneous networks by aggregating information from neighbouring nodes. As Figure 1(B) shows, each piRNA and disease can be regarded as bule and green node respectively, and the association between them can be represented by the integrated similarity matrix. The GCN takes this integrated similarity matrix as input and performs graph convolution operations to extract features that capture the dependencies between piRNAs and diseases.

In GCN, the output of $l$th layer is considered as the input of $(l+1)$-th layer to capture higher dimensional features; the node embedding of $(l+1)$-th layer is as follows: 


(13) \begin{align*}& \boldsymbol{H}^{l+1}=\sigma\left({\boldsymbol{D}}^{-\frac{1}{2}} {\boldsymbol{A}_{h}} {\boldsymbol{D}}^{-\frac{1}{2}} \boldsymbol{H}^{l} \boldsymbol{W}^{l}\right),\end{align*}

where $\boldsymbol{A}_{h}$ is obtained by Equation 2, $\boldsymbol{D}$ is the degree matrix of $\boldsymbol{A}$, $\boldsymbol{H}^{l}$ is the node embedding of the $l$th layer and $\boldsymbol{W}^{l}$ is the trainable weight matrix. $\sigma $ is the activation function ReLU. The initial embedding $\boldsymbol{H}^{0}$ is concatenated by the embedding vectors of piRNAs and diseases; the initial embedding of piRNA is obtained by the method described in piRNA sequence embedding section; the initial embedding of disease is equal to the integrated disease similarity matrix $\boldsymbol{S}_{d}$. $\boldsymbol{H}^{l}$ is combined with the embedding of piRNAs and diseases; the first $m$ rows are the embedding of piRNAs, the last $n$ rows are the embedding of diseases and $m$ and $n$ are the numbers of piRNAs and diseases, respectively.

### Prediction using attention mechanism

To improve the performance of the GCN model, an attention mechanism is introduced. The attention mechanism shown in Figure 1(C) helps the model to focus on the most relevant pairs between piRNAs and diseases. It assigns different weights to nodes during the aggregation process, based on their importance and relevance to the target node. The more important and relevant a piRNA and disease node is, the higher the attention score it will receive. This helps the model prioritize and emphasize the most informative features when predicting PDAs. By incorporating the attention mechanism, the model becomes more sensitive to important features and can better capture the complex relationships between piRNAs and diseases.

**Figure 2:**
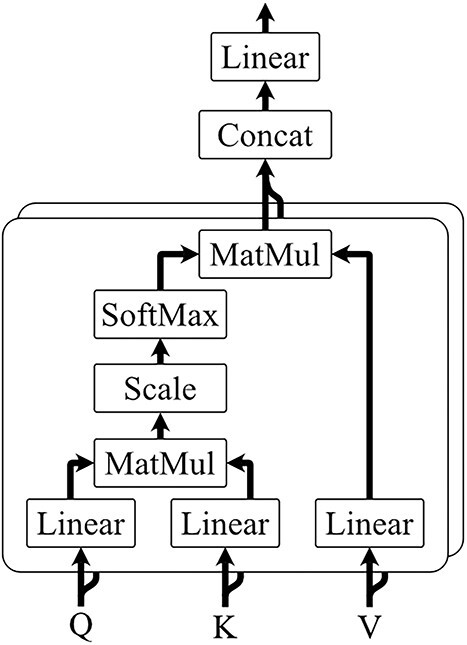
Multi-head attention.

Figure 2 gives a schematic view of the attention mechanism. There are three inputs for multi-head attention, query matrix $\boldsymbol{Q}$, key matrix $\boldsymbol{K}$ and value matrix $\boldsymbol{V}$. For the encoder that extracts piRNA features, the $\boldsymbol{Q}$ matrix is the embedding of piRNAs obtained by GCN and the $\boldsymbol{K}$, $\boldsymbol{V}$ matrices are the embedding of diseases. For the encoder that extracts disease features, it is opposite to this. $\boldsymbol{Q}$ and $\boldsymbol{K}$ are used to calculate the attention score, and $\boldsymbol{V}$ represents the feature of diseases or piRNAs. The output of scaled dot-product attention can be calculated as 


(14) \begin{align*}& \operatorname{Attention}(\boldsymbol{Q}^{\prime}, \boldsymbol{K}^{\prime}, \boldsymbol{V}^{\prime})=\operatorname{softmax}\left(\frac{\boldsymbol{Q}^{\prime} {\boldsymbol{K}^{\prime}}^{\mathsf{T}}}{\sqrt{d_{k}}}\right) \boldsymbol{V}^{\prime},\end{align*}

where 


(15) \begin{align*}& \begin{aligned}\boldsymbol{Q}^{\prime}&=\boldsymbol{QW}^{Q}\\ \boldsymbol{K}^{\prime}&=\boldsymbol{KW}^{K}\\ \boldsymbol{V}^{\prime}&=\boldsymbol{VW}^{V}\end{aligned}\end{align*}

$\boldsymbol W^{Q}$ , $\boldsymbol W^{K}$ and $\boldsymbol W^{V}$ are trainable parameter matrices, and $d_{k}$ is the dimension of the feature vector.

Multi-head attention combines multiple attention heads to further enhance the model’s ability to capture diverse and complex interactions between piRNAs and diseases. The outputs of each head are concatenated together:


(16) \begin{align*}& \begin{aligned} \operatorname{MultiHead}(\boldsymbol{Q}, \boldsymbol{K}, \boldsymbol{V}) =\operatorname{Concat}\left(\operatorname{head}_{1}, \ldots, \operatorname{head}_{\mathrm{h}}\right) \boldsymbol{W}^{O} \\ \text{ where} \operatorname{head}_{\mathrm{i}} =\operatorname{Attention}\left(\boldsymbol{Q W}_{i}^{Q}, \boldsymbol{K W}_{i}^{K}, \boldsymbol{V W}_{i}^{V}\right)\\ \end{aligned},\end{align*}

where $\mathrm{h}$ is the number of heads, which is set to 2 by default. Each row of $\operatorname{MultiHead}(\boldsymbol Q, \boldsymbol K, \boldsymbol V)$ represents the embedding of each piRNA or disease, which is combined by feature vectors based on different attention scores. The following addition and normalization blocks aggregate original features and integrated features from the attention mechanism; the association scores between each piRNA and disease is the inner product of their feature vectors.

### Selecting reliable negative associations using positive unlabelled leaning

In our dataset, unlabelled associations can contain hidden positives that were not labelled as such due to various reasons such as cost or technological limitation, so it is unsafe to assume that all unlabelled examples are negative, especially if positive examples are rare compared with all possible examples. Treating all unlabelled examples as negatives can introduce a bias in the learning process because the negative samples are not truly a representative of the negative class. This can skew the distribution of the dataset and cause the learned model to perform poorly.

So, the PU learning technique is introduced to identify reliable negative associations to help avoid the issues associated with treating all unlabelled data as negative associations [34]. Many techniques have been proposed to address this problem. iPiDi-PUL, iPiDi-sHN and piRDA use the bagging strategy to select high-quality negative associations [15–17]. In this study, we combined three different methods including PU bagging, two-step and spy technique to select reliable negative associations, and compared their performance in the prediction task. The steps of these three PUL method are as follows.

- Spy technique The main idea of the spy technique is to intentionally plant some known positive examples as spies within the set of unlabelled associations, then train an initial classifier under the assumption that all unlabelled examples are negative. All of the unidentified negative associations that possess a lower posterior probability than any undercover operative are deemed trustworthy negative associations.
  1. Embed PDAs The feature of each association will be used in identifying reliable negatives. The features of the association between the $i$th piRNA and the $j$th disease can be represented as follows:

(17) \begin{align*}& \boldsymbol{F}_{i, j}=\left[\begin{array}{ll} \boldsymbol{S}_{p}(i,) & \boldsymbol{S}_{d}(j,) \end{array}\right],\end{align*}

where $\boldsymbol{S}_{p}(i,)$ is the feature vector representing the similarity between the $i$th piRNA and other piRNAs. $\boldsymbol{S}_{d}(j,)$ is the feature vector representing the similarity between the $j$th disease and other diseases.
2. Select spies Randomly select 5% of the labelled positive associations and hide their labels, treating them as spies among the unlabelled data.
3. Train a classifier Train a Random Forest classifier with 500 estimators using the remaining labelled positives as positive examples and all other examples (including both actual unlabelled and spies) as negative examples.
4. Classify spies Use the trained Random Forest classifier to classify all unlabelled examples, including the spies.
5. Identify reliable negatives Identify reliable negative associations by selecting those unlabelled associations with a lower posterior probability of being positive than any of the spies.

- PU bagging The main idea of PU bagging is to use multiple relatively accurate small classifiers to classify unlabelled associations, and those with lower average scores are considered as reliable negative associations.
  1. Embed PDAs Embedding each association using the similarity between the nodes expressed in Equation 17.
  2. Train classifiers Randomly select a portion of unlabelled associations as negative samples with the same size as positive samples and use them with positive samples to train a random forest classifier. Repeat this step 30 times.
  3. Classify unlabelled associations Predict the probability of the remaining unselected and unlabelled samples being positive, iterate five times, and take the average as the final probability associated with being positive.
  4. Identify reliable negatives Sorting the prediction scores of unlabelled samples and dividing them into three clusters. The second cluster, made up of PDA samples, was considered a reliable negative sample set. This improved the training and led to significant performance gains [34].
- Two-Step The main idea of two-step is to obtain a more reliable classifier by updating, so that a more reliable classification can be obtained.
  1. Embed PDAs Embedding each association using the similarity between the nodes expressed in Equation 17.
  2. Train and classify All unlabelled samples are treated as negative samples and used to train a random forest classifier. Use this classifier to classify all associations.
  3. Update and retrain Associations with scores higher than the highest positive association score are updated as positive samples, while associations with scores lower than the lowest positive association score are updated as negative samples. Retrain a classifier.
  4. Iteration Use the new classifier to classify all samples and repeat the above steps five times.
  5. Identify reliable negatives All negative samples are considered reliable negative samples.

The final reliable negative associations set is the union of reliable negative associations obtained from these three methods.

## RESULT

### Performance evaluation

The 5-fold cross-validation method is used to evaluate the performance of the model. The known PDA set $\mathbb{S}^{+}$ and unlabelled association set $\mathbb{S}^{\text{U}}$ can be divided into five subsets with the same size as follows: 


(18) \begin{align*}& \begin{aligned} \mathbb{S}^{+} &= \mathbb{S}^{+}_{1} \cup \mathbb{S}^{+}_{2} \cup \mathbb{S}^{+}_{3} \cup \mathbb{S}^{+}_{4} \cup \mathbb{S}^{+}_{5}\\ \mathbb{S}^{\text{U}} &= \mathbb{S}^{\text{U}}_{1} \cup \mathbb{S}^{\text{U}}_{2} \cup \mathbb{S}^{\text{U}}_{3} \cup \mathbb{S}^{\text{U}}_{4} \cup \mathbb{S}^{\text{U}}_{5} \end{aligned}\end{align*}

The training set and test set can be denoted as follows: 


(19) \begin{align*}& \begin{aligned} \mathbb{S}^{\text{train}}_{i} &= \complement_{\mathbb{S}^{+}}{\mathbb{S}^{+}_{i}} \cup \mathbb{S}^{-}_{i}\\ \mathbb{S}^{\text{test}}_{i} &= \mathbb{S}^{+}_{i} \cup \mathbb{S}^{\text{U}}_{i} \end{aligned},\end{align*}

where $i \in \{1,2,3,4,5\}$, the reliable negative association set $\mathbb{S}^{-}_{i}$ is obtained from $\complement _{\mathbb{S}^{+}}{\mathbb{S}^{+}_{i}} \cup \complement _{\mathbb{S}^{\text{U}}}{\mathbb{S}^{\text{U}}_{i}}$ by Selecting reliable negative associations using positive unlabelled leaning and $\complement $ represents the complement operation. It should be pointed out that for each fold, the GIP kernel similarity matrix $\boldsymbol{S}_{p}^{\text{GIP}}$ and $\boldsymbol{S}_{d}^{\text{GIP}}$ need to be recalculated based on the new training set.

The area under the receiver operating characteristics curve (AUC), the area under the precision recall curve (AUPR) and F1-score are used to evaluate the performance of the prediction model for tackling the imbalanced issue [35–37]. Considering that the value of accuracy, recall, precision, specificity, sensitivity and Matthews correlation coefficient will vary according to the classification threshold, and there is currently no standardized method for selecting thresholds in the association prediction task [15, 17, 19, 22, 23], these indicators are not used when measuring the performance of models.

The rank of positive predictions can also reflect the performance of the model [16]. The prediction scores of all the test piRNA–disease pairs are ranked in descending order and measured using the rank index. The rank index is calculated as follows: 


(20) \begin{align*}& \text{rank index}=\frac{1}{\left|\mathbb{S}_{\text{test}}^{+}\right|} \sum_{a \in \mathbb{S}_{\text{test}}^{+}} \frac{r_{a}}{\left|\mathbb{S}_{\text{test}}\right|},\end{align*}

where $\left | \mathbb{S}_{\text{test}}^{+} \right |$ is the number of all known PDAs in the test subset $\mathbb{S}_{\text{test}}^{+}$, and $\left | \mathbb{S}_{\text{test}} \right |$ is the number of all piRNA–disease pairs in the test set $\mathbb{S}_{\text{test}}^{+}$. $a$ is an association in positive test subset $\mathbb{S}_{\text{test}}^{+}$, and $r_{a}$ represents its rank position of all positive test subset. A lower value of the rank index indicates a better performance of the model.

### Comparison with state-of-the-art methods

We compared the performance of PUTransGCN with six state-of-the-art predictors on two datasets, including ETGPDA [19], iPiDi-PUL [16], iPiDA-GCN [22], iPiDA-SWGCN [23], iPiDA-GBNN [21] and piRDA [17]. The configurations for model architectures were reproduced in PyTorch(version 2.1.2), drawing upon the details provided in their respective publications and GitHub repositories. If their implementation was executed utilizing the PyTorch framework, the code was reused as extensively as feasible. For the models that did not release their code, the parameters of these models are used as stated in their publications. All the data and reproduced code are available at https://github.com/chenqiuhao/PUTransGCN.

The results in Table 1 and Table 2 are obtained by calculating the average and variance of the last five iterations for all five folds on MNDR v4.0 and piRDisease v1.0 dataset, respectively. On these two datasets, PUTransGCN outperforms other art-of-the-state methods in terms of rank index, AUC and AUPR. Applying the Mann–Whitney U test to the score obtained by these methods, the *P*-values between PUTransGCN and other methods are 2.87e-14 (ETGPDA), 6.40e-19 (iPiDi-PUL[DT]), 1.47e-22 (iPiDi-PUL[SVM]), 3.54e-14 (iPiDi-PUL[RF]), 1.62e-20 (iPiDA-GCN), 8.14e-20 (iPiDA-SWGCN), 1.78e-20 (iPiDA-GBNN) and 1.84e-16 (piRDA), which indicates that the performance of PUTransGCN differs from that of other methods in statistics.

**Table 1:** Performance comparison among different methods on MNDR v4.0

	Rank index	AUC	AUPR
ETGPDA	0.116$\pm $0.018	0.916$\pm $0.020	0.417$\pm $0.148
iPiDi-PUL(DT)	0.444$\pm $0.021	0.569$\pm $0.026	0.117$\pm $0.008
iPiDi-PUL(SVM)	0.292$\pm $0.01	0.725$\pm $0.011	0.133$\pm $0.006
iPiDi-PUL(RF)	0.238$\pm $0.016	0.784$\pm $0.018	0.165$\pm $0.009
iPiDA-GCN	0.145$\pm $0.057	0.885$\pm $0.062	0.427$\pm $0.040
iPiDA-SWGCN	0.113$\pm $0.004	0.920$\pm $0.004	0.468$\pm $0.015
iPiDA-GBNN	0.153$\pm $0.010	0.879$\pm $0.014	0.461$\pm $0.114
piRDA	0.120$\pm $0.003	0.912$\pm $0.004	0.338$\pm $0.007
PUTransGCN$^{1}$	**0.103$\pm $0.006**	**0.930$\pm $0.007**	**0.598$\pm $0.032**

$^{1}$ Results obtained via combined PUL method, with the parameters being a piRNA embedding dimension of 128, the number of attention heads being two and spy percentage of 5%.

**Table 2:** Performance comparison among different methods on piRDisease v1.0

	Rank index	AUC	AUPR
ETGPDA	0.093$\pm $0.010	0.931$\pm $0.011	0.449$\pm $0.140
iPiDi-PUL(DT)	0.386$\pm $0.058	0.622$\pm $0.060	0.091$\pm $0.015
iPiDi-PUL(SVM)	0.298$\pm $0.011	0.719$\pm $0.012	0.130$\pm $0.007
iPiDi-PUL(RF)	0.264$\pm $0.021	0.758$\pm $0.023	0.183$\pm $0.018
iPiDA-GCN	0.101$\pm $0.014	0.922$\pm $0.015	0.428$\pm $0.072
iPiDA-SWGCN	0.095$\pm $0.015	0.929$\pm $0.016	0.451$\pm $0.030
iPiDA-GBNN	0.209$\pm $0.076	0.807$\pm $0.082	0.328$\pm $0.071
piRDA	0.090$\pm $0.004	0.933$\pm $0.004	0.359$\pm $0.011
PUTransGCN$^{1}$	**0.074$\pm $0.006**	**0.950$\pm $0.006**	**0.679$\pm $0.017**

$^{1}$ Results obtained via combined PUL method, with the parameters being a piRNA embedding dimension of 128, the number of attention heads being two and spy percentage of 5%.

### Ablation study

To better evaluate the improvement of the PUL method in our proposed model, several ablation experiments were carried out. In this experiment, we compared the performance of these three PUL methods including PU bagging, two-step, spy technique, their combination and do not use the PUL method.

The results in Table 3 and Table 4 are obtained by calculating the average and variance of the last five iterations for all five folds. As shown in tables, the combined method performs better in terms of rank index and AUC than using them alone or not using them. On different datasets, different methods show varying degrees of improvement. For example, the performance of spy technique is best on MNDR v4.0, while the two-step method shows the most significant improvement on piRDisease v1.0. Therefore, combining these three methods can better integrate the advantages of different methods and improve overall performance.

**Table 3:** Performance comparison among using different PU learning methods on MNDR v4.0

	Rank index	AUC	AUPR
PUTransGCN(no PU learning)	0.119$\pm $0.042	0.913$\pm $0.045	0.522$\pm $0.148
PUTransGCN(PU bagging)	0.121$\pm $0.002	0.909$\pm $0.002	**0.649$\pm $0.003**
PUTransGCN(two-step)	0.121$\pm $0.002	0.909$\pm $0.003	**0.649$\pm $0.003**
PUTransGCN(spy)	0.111$\pm $0.006	0.921$\pm $0.007	0.598$\pm $0.032
PUTransGCN(combined)	**0.103$\pm $0.006**	**0.930$\pm $0.007**	0.598$\pm $0.032

**Table 4:** Performance comparison among using different PU learning methods on piRDisease v1.0

	Rank index	AUC	AUPR
PUTransGCN(no PU learning)	0.093$\pm $0.017	0.931$\pm $0.018	0.438$\pm $0.077
PUTransGCN(PU bagging)	0.120$\pm $0.005	0.926$\pm $0.003	0.623$\pm $0.012
PUTransGCN(two-step)	0.075$\pm $0.004	0.949$\pm $0.004	0.679$\pm $0.010
PUTransGCN(spy)	0.124$\pm $0.006	0.932$\pm $0.009	0.566$\pm $0.023
PUTransGCN(combined)	**0.074$\pm $0.006**	**0.950$\pm $0.006**	**0.679$\pm $0.017**

### The influence of the percentage of spy

In the second step of applying the spy technique, a certain proportion of unlabelled associations is selected as spies. More proportion of spy implies more unlabelled associations would be considered as negative, but there may be true positive within these negative associations that could interfere with the model. Having fewer proportions of spy can make the selected negative associations more reliable, but having fewer negative associations can affect the generalizability of the model. Therefore, finding a balance point for the proportion of the spy is crucial.

In this section, we investigated the impact of different proportions of spy on the performance of combined methods. As shown in Table 5, when the percentage of spy reaches 5%, the AUC value of the combined method reaches a maximum of 0.93, and the AUC values on both sides of 5% drop significantly to around 0.923. Meanwhile, the rank index value is at its minimum when the spy ratio is 5%, which means that the real correlation is ranked very high in the predicted results.

**Table 5:** Performance comparison among using different percentage of spy within combined method on MNDR v4.0

PUTransGCN combined	Rank index	AUC	AUPR
1% spy	0.108$\pm $0.008	0.924$\pm $0.008	0.593$\pm $0.033
2% spy	0.109$\pm $0.010	0.923$\pm $0.011	0.576$\pm $0.067
3% spy	0.106$\pm $0.013	0.926$\pm $0.015	0.598$\pm $0.027
4% spy	0.104$\pm $0.004	0.929$\pm $0.004	0.581$\pm $0.013
5% spy	**0.103$\pm $0.006**	**0.930$\pm $0.007**	0.598$\pm $0.032
6% spy	0.104$\pm $0.010	0.928$\pm $0.011	0.583$\pm $0.019
7% spy	0.109$\pm $0.009	0.923$\pm $0.011	**0.610$\pm $0.036**
8% spy	0.110$\pm $0.009	0.922$\pm $0.010	0.606$\pm $0.039
9% spy	0.106$\pm $0.006	0.926$\pm $0.007	0.595$\pm $0.034
10% spy	0.107$\pm $0.007	0.925$\pm $0.008	0.597$\pm $0.038

### Case study

To verify whether the model can accurately predict associations beyond the dataset, we trained it with all the data and made predictions for all diseases. We selected four diseases from them and searched for the accuracy of positive associations predicted by the model. Table 6 shows some of the prediction results for these diseases. It can be seen that out of 20 associations, 18 have been experimentally confirmed. For instance, in renal carcinoma cells, the expression of DQ597483 is down-regulated to normal cells [38]. On the other hand, DQ597397 is up-regulated in Renal Carcinoma Cell [38]. Similarly, DQ570326 and DQ592957 demonstrate down-regulation in Parkinson’s disease derived neuronal cells [39]. Moreover, DQ569948, FR338565 and DQ594531 exhibit differential expression in lung tumour tissue compared with the normal human lung tissue [40]. Furthermore, in neural cells from Alzheimer’s disease-affected brain, DQ596377 is up-regulated and exhibits 11.38 fold higher expression in neural cells compared with normal human brain cells [41]. The remaining expression levels of piRNA shown in the table are different from those of normal cells.

**Table 6:** The top five piRNAs associated with different diseases predicted by PUTransGCN, and its prediction result obtained by other four methods

Disease	piRNA	Evidence	PUTransGCN	ETGPDA	iPiDi-PUL		
					DT	SVM	RF
Renal Cell Carcinoma	DQ597483	PMID:25998508	✓		✓	✓	✓
	DQ597397	PMID:25998508	✓	✓		✓	✓
	DQ581939	PMID:25998508	✓		✓	✓	✓
	DQ582873	PMID:25998508	✓				
	DQ583291	PMID:25998508	✓		✓		
Parkinson Disease	DQ570326	PMID:29986767	✓				
	DQ592957	PMID:29986767	✓	✓		✓	
	DQ573919	PMID:29986767	✓	✓			
	DQ571691	PMID:29986767	✓	✓			
	DQ597479	PMID:29986767	✓	✓		✓	✓
Lung Cancer	DQ569948	PMID:28423657	✓		✓		
	hsa:piR_000823	Unconfirmed	✓		✓	✓	✓
	FR338565	PMID:28423657	✓	✓	✓	✓	✓
	DQ594531	PMID:28423657	✓	✓		✓	✓
	FR111727	Unconfirmed	✓	✓		✓	✓
Alzheimer Disease	DQ596377	PMID:28127595	✓	✓		✓	✓
	PIR36772	PMID:28127595	✓	✓	✓	✓	✓
	DQ598159	PMID:28127595	✓	✓	✓	✓	✓
	DQ576200	PMID:28127595	✓	✓		✓	✓
	DQ593431	PMID:28127595	✓	✓		✓	✓

In order to compare the predictive abilities of these models, we also examined the predictions of other models on these 20 associated cases. If the prediction score of association ranks in the top 10 percent within its corresponding disease, it is considered as an positive association; otherwise, it is considered as negative. iPiDA-GCN, iPiDA-SWGCN, iPiDA-GBNN and piRDA did not predict any of these 20 associations and therefore is not listed in the table. Among these 20 associations, ETGPDA predicted 13, iPiDi-PUL(DT) predicted 8, iPiDi-PUL(SVM) predicted 14 and iPiDi-PUL(RF) predicted 13. PUTransGCN can predict more associations for these 20 cases. These findings suggest that PUTransGCN has the potential to identify new PDAs, with unconfirmed associations serving as candidate targets for future biological experiments.

## CONCLUSION

In this study, a deep learning predictor called PUTransGCN based on PUL was proposed to identify PDAs. This model integrated heterogeneous graph neural networks with the attention mechanism to uncover potential associations. Additionally, to address the imbalance and sparsity in the dataset, the spy technique was utilized to recognize reliable negatives.

The performance of PUTransGCN was evaluated through 5-fold cross-validation on the MNDR v4.0 and piRDisease v1.0 database. PUTransGCN outperforms other six state-of-the-art methods in terms of rank index, AUC and AUPR. Ablation experiments demonstrated that the combined PUL method led to considerable enhancements over non-use or using the single PUL approach. Furthermore, case studies on diseases like lung cancer and Parkinson’s correctly recovered differentially expressed piRNAs from literature, which indicates the model has the ability to predict potential correlations and can provide references for future medical testing.

However, there are still some limitations. First, three PUL methods are used; each one needs to be classified with a classifier for all associated categories, which is time consuming. Also, only a small portion of the associations have been discovered in the laboratory; the amount of data will definitely increase in the future. Transformer has the drawback of high computational demands and could encounter challenges in keeping pace with current trends. Diseases are not only associated with piRNA, but also with micro RNA and long non-coding RNA. Currently, there is a lack of a unified model that can perform well in predicting the association between all these ncRNAs and diseases simultaneously. In the future, we would like to generalize this model to datasets of other types of ncRNA. Moreover, we can also conduct validation experiments on the associations predicted by our model.

**Key Points**

- We use word2vec and TextCNN to embed the piRNA sequence, which not only includes information of the entire sequence but also information of subsequences.
- Three PUL methods including PU bagging, two-step and spy technique are applied to select reliable negative associations for addressing the problem of imbalanced dataset.
- Two attention modules are applied to integrate the feature of piRNAs and diseases. piRNAs and diseases act as query and key to calculate the attention score between them, which can integrate their information throughly.
- The experimental results on benchmark datasets show that PUTransGCN outperforms other six state-of-the-art approaches. Case study shows that PUTransGCN has the potential to identify new PDAs.

## Data Availability

The code and raw data are released to Zenodo with identifier 10688775. The source code of PUTransGCN are available at https://github.com/chenqiuhao/PUTransGCN.

## Author Biographies

**Qiuhao Chen** is a researcher in Institute of Bioinformatics at Harbin Institute of Technology. His expertise is bioinformatics.

**Liyuan Zhang** is a PhD student in Department of Computer Science at Harbin Institute of Technology. Her expertise is bioinformatics.

**Yaojia Liu** is a bachelor student in Department of Computer Science at Harbin Institute of Technology. His expertise is bioinformatics.

**Zhonghao Qin** is a PhD student in State Key Laboratory of Robotics and System at Harbin Institute of Technology. His expertise is machine learning.

**Tianyi Zhao** is a professor in school of Medicine and Heath at Harbin Institute of Technology. His expertise is bioinformatics and machine learning.
